# Epigenetic signatures of gestational diabetes mellitus on cord blood methylation

**DOI:** 10.1186/s13148-017-0329-3

**Published:** 2017-03-27

**Authors:** Larissa Haertle, Nady El Hajj, Marcus Dittrich, Tobias Müller, Indrajit Nanda, Harald Lehnen, Thomas Haaf

**Affiliations:** 1 Institute of Human Genetics, Julius-Maximilians-Universität Würzburg, Biozentrum, Am Hubland, 97074 Würzburg, Germany; 2 Department of Bioinformatics, Julius Maximilians University, 97074 Würzburg, Germany; 3 Department of Gynecology and Obstetrics, Municipal Clinics, 41239 Moenchengladbach, Germany

**Keywords:** DNA methylation, Fetal cord blood, Fetal programming, Gestational diabetes mellitus, Insulin treatment

## Abstract

**Background:**

Intrauterine exposure to gestational diabetes mellitus (GDM) confers a lifelong increased risk for metabolic and other complex disorders to the offspring. GDM-induced epigenetic modifications modulating gene regulation and persisting into later life are generally assumed to mediate these elevated disease susceptibilities. To identify candidate genes for fetal programming, we compared genome-wide methylation patterns of fetal cord bloods (FCBs) from GDM and control pregnancies.

**Methods and results:**

Using Illumina’s 450K methylation arrays and following correction for multiple testing, 65 CpG sites (52 associated with genes) displayed significant methylation differences between GDM and control samples. Four candidate genes, *ATP5A1*, *MFAP4*, *PRKCH*, and *SLC17A4*, from our methylation screen and one, *HIF3A*, from the literature were validated by bisulfite pyrosequencing. The effects remained significant after adjustment for the confounding factors maternal BMI, gestational week, and fetal sex in a multivariate regression model. In general, GDM effects on FCB methylation were more pronounced in women with insulin-dependent GDM who had a more severe metabolic phenotype than women with dietetically treated GDM.

**Conclusions:**

Our study supports an association between maternal GDM and the epigenetic status of the exposed offspring. Consistent with a multifactorial disease model, the observed FCB methylation changes are of small effect size but affect multiple genes/loci. The identified genes are primary candidates for transmitting GDM effects to the next generation. They also may provide useful biomarkers for the diagnosis, prognosis, and treatment of adverse prenatal exposures.

**Electronic supplementary material:**

The online version of this article (doi:10.1186/s13148-017-0329-3) contains supplementary material, which is available to authorized users.

## Background

The “developmental origins of health and disease (DOHAD)” or Barker hypothesis associates adverse environmental exposures in the periconceptional and/or intrauterine period with lifelong increased morbidity for metabolic, cardiovascular, and other complex diseases [1, 2]. A large number of studies provided convincing evidence that both fetomaternal under- and overnutrition negatively influence the metabolic phenotype of the exposed individuals in later life [3, 4]. The prevalence of obesity and of women developing gestational diabetes mellitus (GDM) is increasing worldwide [5, 6]. Depending on ethnicity and diagnostic criteria, GDM affects 2 to >10% of all pregnancies.

Changes in lifestyle (overnutrition and physical inactivity) and genetic risk factors [7, 8] alone cannot explain the current GDM epidemics. GDM develops during pregnancy (usually in late second trimester) when the maternal insulin production can no longer cope with increasing adiposity and insulin resistance (due to increased placental lactogen, estrogen, and prolactin) [9, 10]. It results in fetal overnutrition (with glucose, amino acids, lipids, and fatty acids) and fetal hyperinsulinism, which may cause medical problems (macrosomia, organomegaly, and neonatal hypoglycemia) in the perinatal period. In addition, the adverse intrauterine environment may lead to persistent developmental malprogramming of the metabolism [11]. The offspring of GDM mothers have increased risks of developing obesity, type 2 diabetes, and cardiovasular disease [12–15]. Moreover, GDM exposure has been associated with autism spectrum disorder and long-term neuropsychiatric morbidity [16]. Studies of Pima Indian siblings discordant for exposure to GDM indicate that in addition to shared risk alleles, the increased lifelong disease risk is at least partially mediated by the hyperglycemic intrauterine environment [17]. The most likely mechanisms for translating the effects of intrauterine GDM exposure into disease susceptibility is epigenetic dysregulation of metabolic, cardiovascular, and neuroendocrine pathways [18–20].

Epigenetic mechanisms control gene expression patterns without altering the DNA sequence. The most thoroughly studied epigenetic modification is DNA methylation, more precisely methylation of cytosine carbon 5 at cytosine phosphate guanine (CpG) dinucleotides. DNA methylation patterns are transmitted to daughter cells during somatic cell division and perhaps also from one generation to the next. Promoter methylation during development, differentiation, or disease processes leads to an inactive chromatin structure and gene silencing. In contrast, gene body methylation is usually associated with active genes [21–23]. One important hallmark of DNA methylation patterns is their enormous plasticity during development and in response to environmental factors [24, 25]. Epigenetic modifications are primary candidates for mediating the persistent effects of an adverse intrauterine environment on the metabolism of the exposed individual.

Mass spectrometry revealed an increased global DNA methylation in placenta of GDM mothers [26]. Candidate gene studies have identified a number of differentially methylated genes in fetal tissues of babies from GDM mothers, including the fat-cell hormones leptin (*LEP*) and adiponectin (*ADIPOQ*), which are involved in regulation of energy metabolism and body weight [27, 28], the ATP-binding cassette transporter *ABCA1*, a major regulator of cellular cholesterol [29], the glucose transporters *SLC2A1/GLUT1* and *SLC2A3/GLUT3* [30], and the imprinted gene *MEST*, which plays a role in adipositas development [31]. In addition, there are already several genome-wide methylation studies in the offspring of GDM mothers [32–36], which have led to the identification of functional networks (various metabolic disease pathways and processes, cell growth and death regulation, endocytosis, inflammatory response, MAPK signaling, MODY, NOTCH signaling, type 2 diabetes) which are epigenetically programmed through GDM exposure. It is interesting to note that despite comparable sample sizes and study design, the number of identified loci with genome-wide significance ranged from none [32] to over thousand [35] and there is limited overlap between the identified genes and pathways. The GDM-susceptible genes that have been discovered so far may represent only the tip of the iceberg and also need to be replicated in independent studies. Here, we performed a 450K methylation array screen on cord bloods from GDM mothers and matched controls. To minimize the effects of confounding factors, all study subjects (the vast majority of them Caucasians) were recruited from a single obstetric clinic. Unlike other studies, diabetes during pregnancy was very well controlled. We distinguished between insulin-treated GDM (I-GDM) and dietetically treated GDM (D-GDM), assuming that I-GDM represents a more severe phenotype and more adverse fetal exposure.

## Methods

### Study subjects and DNA samples

Umbilical cord bloods from newborns (singletons) of 105 mothers with I-GDM, 88 with D-GDM, and 120 controls without GDM were collected by obstetricians at the Municipal Clinics, Moenchengladbach, Germany. Blood samples were immediately frozen at −80 °C until further use. Genomic DNA was isolated with the FlexiGene DNA kit (Qiagen, Hilden, Germany) and bisulfite conversion performed with the EpiTect Fast 96 kit (Qiagen).

GDM was diagnosed between gestational weeks 24 and 27 by an elevated fasting (for 8-12 h) plasma glucose (>5.1 mmol/l) and a pathological oral glucose tolerance test (>10 mmol/l at 1 h and/or >8.5 mmol/l at 2 h after drinking a solution with 75-g glucose). Following diagnosis, women received dietary counselling by a diabetologist. According to the recommendations of the German Society of Gynecology and Obstetrics (DGGG) and the American Diabetes Association (ADA), they were put on a diet consisting of approximately 45% carbohydrate, 30–35% fat, and up to 20% protein. Protein intake was limited to approximately 0.8 g/kg body weight. The patients were not allowed to fast. If dietary treatment did not decrease glucose (<5.1 mmol/l after fasting, <7.8 mmol/l at 1 h, and <6.7 mmol/l at 2 h after meals) and HbA1C levels (<6%), patients were treated with the basis bolus insulin and rarely insulin pump therapy.

### Microarray analysis

Two independent methylation array data sets (NCBI GEO accession no. GSE88929) were generated. Data set A represents 20 I-GDM and 18 control samples and data set B 24 I-GDM, 24 D-GDM, and 46 control samples (Table 1). After bisulfite conversion, the 38 samples of data set A and the 94 samples of data set B were whole-genome amplified, enzymatically fragmented, and hybridized to 4 and 8 Illumina HumanMethylation450 BeadChips, respectively, according to the manufacturer’s protocol (Illumina, San Diego, CA, USA). The arrays were scanned with an Illumina iScan. Microarray data were exported as idat files and analyzed using the statistical software package R (version 3.2.2) and the BioConductor platform (version 3.2). Preprocessing has been performed using the infrastructure implemented in the minfi [37] and watermelon [38] packages. First, sites with low signal quality (beadcount <3 and detection *p* value >0.05) were filtered and sites overlapping known SNPs removed. Furthermore, probes on the sex chromosomes were excluded, leaving a total number of 452,932 probes (cohort A) and 455,307 probes (cohort B), respectively, for subsequent analyses (out of >485,000 CpGs on the chip covering 99% of RefSeq genes with promoter, first exon, gene body, 5′ and 3′ UTRs and 96% of CpG islands). Intensity values were normalized using the dasen method as implemented in the watermelon package [38]. To account for potential probe-type effects, an intra-sample normalization procedure (BMIQ) has been applied which corrects for the bias of type 2 probes. Differential methylation analysis has been performed using the moderated *T* test model based on *β* values as implemented in the limma package [39]. All *p* values have been corrected for multiple testing using the Benjamini-Hochberg method [40].

**Table 1:** Clinical parameters of analyzed cohorts and subgroups

Array cohort A	Controls	D-GDM	I-GDM	*p* value
Sample size (*n*)	18		20	
Gestational week	39.1 ± 0.9		39.2 ± 1.3	0.346
Preterm birth (*n*)	0		1	1
Maternal BMI (kg/m^2^)	25.2 ± 3.1		26.3 ± 3.5	0.303
Maternal height (cm)	164.4 ± 6.7		167.9 ± 7.6	0.141
Weight before pregnancy (kg)	69.8 ± 10.8		75.8 ± 12.9	0.133
Weight before birth (kg)	82.0 ± 11.3		88.6 ± 13.3	0.118
HbA1c (%)	n.d.		5.8 ± 0.6	n.d.
Diabetes before pregnancy^a^	0		1 TDM1, 1 TDM2	0.488
Maternal age (years)	29.6 ± 2.8		31.0 ± 4.6	0.259
Parity^b^	7 PP, 6 BP, 5 MP		5 PP, 13 BP, 2 MP	0.965
Spontaneous abortion rate	0.50 ± 0.71		0.15 ± 0.37	0.186
Nicotine consumption^c^	n.d.		17 NS, 3 S	n.d.
Maternal comorbitidies^d^	0		2 T	0.488
Mode of birth^e^	12 UVB, 6 CS		15 UVB, 2 VVB, 3 CS	0.239
Sex of child	8 ♂, 10 ♀		11 ♂, 9 ♀	0.746
Birth weight (g)	3283.6 ± 420.7		3673.3 ± 515.9	0.016
Weight for gestational age^f^	18 AGA		18 AGA, 2 LGA	0.613
Placenta weight (g)	509.4 ± 105.8		566.2 ± 127.0	0.372
Blood pH	7.32 ± 0.05		7.28 ± 0.06	0.019
Array cohort B	Controls	D-GDM	I-GDM	*p* value
Sample size (*n*)	46	24	24	
Gestational week	39.5 ± 1.4	39.1 ± 1.3	38.7 ± 1.5	0.024
Preterm birth (*n*)	2	1	1	1
Maternal BMI (kg/m^2^)	25.3 ± 6.4	25.1 ± 4.1	29.9 ± 7.3	0.018
Maternal height (cm)	166.3 ± 9.4	165.3 ± 6.4	165.9 ± 5.9	0.581
Weight before pregnancy (kg)	71.7 ± 17.9	69.0 ± 10.8	83.6 ± 20.6	0.034
Weight before birth (kg)	86.0 ± 7.7	80.2 ± 10.6	96.7 ± 20.4	0.009
HbA1c (%)	n.d.	5.5 ± 0.4	5.9 ± 0.6	0.020
Diabetes before pregnancy^a^	0	0	3 TDM1, 1 TDM2	0.007
Maternal age (years)	30.3 ± 5.8	31.7 ± 6.1	31.3 ± 4.6	0.526
Parity^b^	28 PP, 10 BP, 8 MP	13 PP, 9 BP, 2 MP	13 PP, 9 BP, 2 MP	0.491
Spontaneous abortion rate	0.46 ± 0.98	0.29 ± 1.04	0.25 ± 0.61	0.370
Nicotine consumption^c^	41 NS, 5 S	23 NS, 1 S	22 NS, 2 S	0.637
Maternal comorbitidies^d^	1 H, 1 T	1 P	1 H	1
Mode of birth^e^	34 UVB, 3 VVB, 9 CS	14 UVB, 1 VVB, 9 CS	18 UVB, 5 CS	0.262
Sex of child	22 ♂, 24 ♀	12 ♂, 12 ♀	12 ♂, 12 ♀	0.978
Birth weight (g)	3391.2 ± 575.9	3396.9 ± 558.4	3465 ± 478.4	0.960
Weight for gestational age^f^	2 SGA, 44 AGA	23 AGA, 1 LGA	23 AGA, 1 LGA	0.183
Placenta weight (g)	n.d	538.4 ± 111.0	552.1 ± 128.0	0.900
Blood pH	7.29 ± 0.10	7.30 ± 0.06	7.26 ± 0.10	0.368
Pyrosequencing cohort	Controls	D-GDM	I-GDM	*p* value
Sample size (*n*)	56	64	61	
Gestational week	39.5 ± 1.4	39.0 ± 1.4	38.8 ± 1.4	0.007
Preterm birth (*n*)	3	3	3	1
Maternal BMI (kg/m^2^)	25.2 ± 5.9	25.7 ± 5.0	31.5 ± 8.1	<0.001
Maternal height (cm)	166.6 ± 9.1	165.4 ± 6.2	166.6 ± 6.5	0.294
Weight before pregnancy (kg)	71.8 ± 16.4	71.1 ± 14.6	89.3 ± 23.4	<0.001
Weight before birth (kg)	85.8 ± 16.5	84.2 ± 15.2	101.2 ± 24.0	<0.001
HbA1c (%)	n.d.	5.5 ± 0.3	5.8 ± 0.4	<0.001
Diabetes before pregnancy^a^	0	0	5 TDM1, 3 TDM2	<0.001
Maternal age (years)	30.4 ± 5.8	31.3 ± 6.1	32.2 ± 5.3	0.243
Parity^b^	33 PP, 13 BP, 10 MP	35 PP, 22 BP, 7 MP	25 PP, 23 BP, 12 MP	0.203
Spontaneous abortion rate	0.52 ± 1.13	0.31 ± 0.77	0.27 ± 0.55	0.846
Nicotine consumption^c^	47 NS, 9 S	57 NS, 7 S	55 NS, 7 S	0.549
Maternal comorbitidies^d^	1 H, 2 T	1 H, 2 P, 1 T	3 H, 1 T	1
Mode of birth^e^	42 UVB, 3 VVB, 11 CS	35 UVB, 1 VVB, 23 CS	43 UVB, 4 VVB, 17 CS	0.273
Sex of child	29 ♂, 27 ♀	25 ♂, 38 ♀	34 ♂, 26 ♀	0.152
Birth weight (g)	3332.2 ± 550.9	3311.6 ± 504.1	3540.0 ± 473.3	0.051
Weight for gestational age^f^	3 SGA, 53 AGA	63 AGA, 1 LGA	56 AGA, 2 LGA	0.064
Placenta weight (g)	n.d.	517.8 ± 99.3	556.3 ± 127.1	0.155
Blood pH	7.29 ± 0.09	7.30 ± 0.06	7.28 ± 0.08	0.593

*n.d.* no data

^a^TDM1 = type 1 diabetes mellitus, TDM2 = type 2 diabetes mellitus

^b^PP = primiparous, BP = biparous, MP = multiparous

^c^NS = non-smoker, S = smoker

^d^H = hypertension, P = preeclampsia, T = thyroid dysfunction

^e^CS = Cesarean section, UVB = unassisted vaginal birth, VVB = ventouse-assisted vaginal birth

^f^SGA = small for gestational age (<3th percentile), AGA = appropriate for gestational age (3rd–97th percentile), LGA = large for gestational age (>97th percentile)

### Bisulfite pyrosequencing

The PyroMark Assay Design 2.0 software (Biotage, Uppsala, Sweden) was used for design of PCR and sequencing primers (Additional file 1: Table S1). Assays were established using the EpiTect PCR Control DNA set (Qiagen) with 0, 25, 50, 75, and 100% methylation. PCR reactions were performed in a total volume of 25 μl using the FastStart Taq DNA polymerase system (Roche Diagnostics, Mannheim, Germany). The 25 μl reaction consisted of 2.5 μl 10× PCR buffer, 20 mM MgCl_2_, 1.0 μl dNTP (10 mM) mix, 10 pmol of forward and reverse primer, 1 IU of FastStart polymerase, 1 μl (approximately 100 ng) bisulfite converted template DNA, and 18.3 μl PCR-grade water. For *SLC17A4*, 2.0 μl template DNA and 17.3 μl water were used.

To reduce technical noise (batch effects), bisulfite conversion and PCR (of D-GDM, I-GDM, and control samples) were performed simultaneously in 96-well microtiter plates. Pyrosequencing was performed on a PyroMark Q96 MD system (Qiagen) using the PyroMark Gold Q96 CDT reagent kit (Qiagen), 10 pmol of sequencing primer, and Pyro Q-CpG software (Qiagen). In our experience, the average methylation difference between technical replicates (including bisulfite conversion, PCR, and pyrosequencing) is approximately 1–2 percentage points. Artificially methylated and unmethylated DNA standards (Qiagen) were included as controls in each pyrosequencing run.

### Statistical testing

Statistical analyses were performed with the statistical software package R (version 3.2.2) and IBM SPSS Statistics 23. The DNA methylation levels at each individual CpG site and the mean of all CpGs for the targeted region were compared between groups using the Mann-Whitney *U* test. To adjust for potential confounding factors, multivariate linear regression models have been used for the analysis of the pyrosequencing data. Potential confounders have been selected based on known and observed factors potentially influencing DNA methylation. The regression coefficients of the final model were adjusted for maternal BMI, gestational age, and fetal sex.

## Results

### Methylation array screens

Our genome-wide study of DNA methylation patterns was based on fetal cord bloods (FCBs) from pregnancies with D-GDM, I-GDM, and without GDM. Clinical parameters of the different cohorts and subgroups are presented in Table 1. The vast majority (>90%) of study subjects were of middle European descent with the remaining few percent from South-Eastern Europe and Turkey. Array cohort A consisted of 20 FCB samples from mothers with I-GDM and 18 controls. Samples were carefully matched for gestational week, fetal sex, maternal BMI, and age. The independent array cohort B consisted of 24 samples from mothers with D-GDM, 24 with I-GDM, and 48 controls. Due to the larger sample size, it was not possible to match for all relevant clinical parameters. Maternal BMI and gestational age differed between groups. In general, women with GDM were managed very well during pregnancy, displaying average HbA1c levels <6%. Only a few women in each group presented with comorbidities such as hypertension, preeclampsia, or thyroid dysfunction. Ten to 17% of women with I-GDM but none with D-GDM group suffered from type 1 or 2 diabetes before pregnancy (Table 1). There were only few preterm births (before 37th week of gestation) and small or large for gestational age (SGA, LGA) babies, respectively. Since white blood counts were not available, the relative proportion of different cell types in the FCBs was estimated from genome-wide methylation profiles using statistical methods [41]. None of the two analyzed cohorts showed a significant difference in cell composition between GDM and control samples (Additional file 2: Figure S1).

Samples of cohort A were hybridized to 4 and cohort B to 8 Illumina HumanMethylation450 BeadChips. We did not find significant differences in global (array CpG) methylation between control, D-GDM, and I-GDM samples in cohort A (*p* = 0.87) and B (*p* = 0.94), respectively. Since methylation levels differ markedly between CpG island (CGI)-related sites and the remaining genome, we performed separate analyses for the array GpG subsets in CGIs, north/south shelfs and shores, and open sea (Additional file 3: Table S2). Although there were no significant between-group differences, it is noteworthy that in cohort B, mean methylation of all targeted CpG subsets was 0.3–1.1 percentage points lower in both the D-GDM and I-GDM groups, compared to controls.

In cohort A, none of the analyzed 452,932 CpGs showed a significant between-group methylation difference after correction for multiple testing. However, the *p* value distribution (histogram) displayed an accumulation of *p* values in the low significance range, indicating the presence of a weak signal in the data set. In cohort B, 11,195 of 455,307 analyzed CpGs exhibited a significant (FDR-adjusted *p* < 0.05) methylation difference between I-GDM and controls and none between D-GDM and controls. Comparative analysis of data sets A and B revealed high concordance (*R* = 0.999, *p* < 2.2E−16) of single CpG methylation values. Both data sets showed a significant correlation of methylation differences (*R* = 0.126; *p* < 2.2E−16) and *T* values (*R* = 0.078; *p* < 2.2E−16) between I-GDM and control samples, consistent with the presence of a shared signal. To extract robust signals, the *p* values of both analyses were combined using order statistics of two uniformly distributed random variables. The first-order statistics revealed 1564 and the more robust second-order statistics 65 significant CpG sites, 52 of which are associated with genes (Table 2). The lack of significant signals in the D-GDM samples may be due to the lower sample size or the less severe metabolic phenotype.

**Table 2:** Array CpGs with significant methylation differences in GDM cord bloods

Array CpG	Gene	Chromosomal location^a^	*β* ^b^ data set A	*β* ^b^ data set B	Adjusted *p* value
cg02943336	*CARD11*	Chr7: 2,959,067	1.08%	1.33%	0.022
cg11449134	CpG island	Chr19: 51,897,791	−0.63%	−0.63%	0.028
cg26001655	*KIAA1530*	Chr4: 1,356,770	1.20%	1.25%	0.028
cg11010397	*AMPH*	Chr7: 38,671,977	−1.37%	−1.81%	0.028
cg22865713	*SLC17A4*	Chr6: 25,779,897	−2.71%	−3.38%	0.028
cg01993865	*DSTN*	Chr20: 17,550,690	−0.60%	−0.78%	0.028
cg18906596	*ANKFY1*	Chr17: 4,151,473	−2.73%	−2.29%	0.028
cg05697697	*XPNPEP1*	Chr10: 111,683,345	0.66%	0.62%	0.028
cg07431064	*CBX7*	Chr22: 39,529,217	0.95%	1.12%	0.028
cg03345925	*ZC3H3*	Chr8: 144,599,347	3.39%	3.83%	0.028
cg06945690	*ZNF167*	Chr3: 44,621,374	2.09%	2.69%	0.031
cg10576992	*SEC24D*	Chr4: 119,662,530	−2.73%	−2.83%	0.031
cg26281025	*HK3*	Chr5: 176,308,046	1.04%	1.38%	0.031
cg08732684	*ATF6B*	Chr6: 32,095,128	1.79%	2.20%	0.035
cg23376861	*ATP5A1*	Chr18: 43,678,713	−3.45%	−4.32%	0.035
cg21143899	*UCK2*	Chr1: 165,866,296	1.42%	1.53%	0.035
cg02683621	North shore	Chr7: 150,100,820	1.15%	1.59%	0.035
cg17921080	Open sea	Chr14: 86,478,932	−1.85%	−2.36%	0.035
cg07689396	*PRKAR1B*	Chr7: 633,050	1.62%	1.42%	0.035
cg08077807	*PRKCH*	Chr14: 62,001,072	−2.23%	−2.53%	0.037
cg19143209	CpG island	Chr9: 19,789,287	−2.43%	−2.74%	0.039
cg15737302	North shelf	Chr11: 118,302,063	−1.90%	−1.85%	0.039
cg19169154	*MFAP4*	Chr17: 19,287,978	1.81%	3.21%	0.039
cg07018980	*GAK*	Chr4: 895,604	1.04%	1.67%	0.040
cg10288510	CpG island	Chr1: 214,158,727	−1.59%	−0.89%	0.040
cg13153307	*SEC16A*	Chr9: 139,368,749	1.89%	1.55%	0.040
cg10778517	*MAD1L1*	Chr7: 2,252,773	0.93%	1.18%	0.040
cg08440349	*ATP2C2*	Chr16: 84,486,704	1.57%	1.33%	0.040
cg01203331	*NOP56*; *SNORD56*, *57*, *86*	Chr20: 2,636,597	1.12%	1.49%	0.040
cg18502630	*PTGDS*	Chr9: 139,871,955	1.44%	1.35%	0.040
cg03246914	*TUBB1*	Chr20: 57,596,113	−3.22%	−3.05%	0.040
cg26706238	*ABCG5*; *ABCG8*	Chr2: 44,066,206	1.35%	1.27%	0.040
cg11703745	*TMCC2*	Chr1: 205,199,293	1.62%	1.87%	0.040
cg19830000	*NELL2*	Chr12: 45,270,312	−0.50%	−0.48%	0.041
cg01205011	*ZNF76*	Chr6: 35,262,113	−2.28%	−2.44%	0.041
cg01955962	CpG island	Chr15: 73,089,536	−0.54%	−0.64%	0.041
cg25871543	*XAB2*	Chr19: 7,686,181	0.91%	1.41%	0.041
cg13984931	North shelf	Chr1: 162,788,667	−2.47%	−2.10%	0.041
cg13706613	*INPP5E*	Chr9: 139,324,927	1.36%	1.10%	0.041
cg03540894	North shelf	Chr12: 133,611,606	−2.46%	−2.84%	0.044
cg01968402	North shore	Chr6: 137,817,775	0.71%	0.58%	0.044
cg26406256	*HERC3*; *NAP1L5*	Chr4: 89,619,393	1.51%	1.83%	0.046
cg04078644	North shelf	Chr3: 155,458,975	−1.36%	−1.69%	0.046
cg08542429	*AGPAT1*	Chr6: 32,139,120	1.49%	1.42%	0.046
cg04514868	*MTA1*	Chr14: 105,931,040	0.92%	1.08%	0.046
cg05536286	*ST8SIA2*	Chr15: 92,972,514	−1.13%	−1.22%	0.046
cg08443019	*OVCA2*; *DPH1*	Chr17: 1,946,299	1.59%	1.41%	0.046
cg24119500	*BAI3*	Chr6: 69,420,780	−1.93%	−1.77%	0.046
cg00273340	*CCDC88B*	Chr 11: 64,112,444	1.22%	1.45%	0.046
cg00730857	*WHSC2*	Chr4: 1,994,281	0.89%	0.90%	0.046
cg12841566	*MADD*	Chr11: 47,296,317	1.29%	0.94%	0.046
cg14088574	*VPS52*	Chr6: 33,234,976	1.17%	1.34%	0.046
cg25927444	*TTC7A*	Chr2: 47,236,103	−2.23%	−1.91%	0.046
cg09244071	*CUX1*	Chr7: 101,768,746	1.00%	1.38%	0.046
cg27509867	Open sea	Chr8: 129,165,585	1.15%	1.42%	0.046
cg26828643	*FAM38A*	Chr16: 88,802,820	1.46%	1.51%	0.048
cg22606873	*PRDM16*	Chr1: 3,144,679	−1.27%	−1.21%	0.048
cg16126178	*AKT1*	Chr14: 105,239,857	1.11%	1.35%	0.048
cg16221240	CpG island	Chr2: 130,970,934	−3.16%	−2.49%	0.049
cg03280063	*GAK*	Chr4: 893,186	1.22%	1.58%	0.049
cg00063535	*TPCN1*	Chr12: 113,729,491	1.35%	1.75%	0.049
cg08144943	*PPM1M*	Chr3: 52,280,702	2.09%	1.91%	0.049
cg17881203	*WDR18*	Chr19: 990,398	0.82%	1.40%	0.049
cg20935025	*NFKBIA*	Chr14: 35,874,013	1.94%	2.70%	0.049
cg14597908	*GNAS*	Chr20: 57,414,960	1.79%	1.35%	0.049

^a^According to the annotation provided by Illumina

^b^Positive *β* difference indicates hypermethylation and negative* β* hypomethylation in the GDM group

Since earlier studies reported a correlation between placental *ADIPOQ* methylation and maternal blood glucose concentration [28], we analyzed the association of HbA1c levels with array CpG methylation in both GDM subgroups (Hb1Ac values were not available for controls). However, neither cohort A nor B displayed any significant sites after multiple testing correction. In addition, we tested the anthropometric surrogate parameters birth weight and gestational age for their association with DNA methylation. In cohort A, none of the analyzed array CpG sites reached genome-wide significance. In cohort B, there were no significant CpG sites for birth weight after multiple testing correction. A small number (823 of 455,307, 0.2%) of CpGs showed a significant association between gestational age and DNA methylation.

### Validation of candidate genes by bisulfite pyrosequencing

Four candidate genes from our methylation array screen, *ATP5A1*, *MFAP4*, *PRKCH*, and *SLC17A4*, were analyzed by bisulfite pyrosequencing in 61 I-GDM, 64 D-GDM, and 56 control samples, including some (mainly control) samples that had been on the array. These genes were selected, because they exhibited between-group methylation differences >2% in both array data sets and were associated with common complications of diabetes in the literature [42–48]. It is noteworthy that maternal BMI, weight before pregnancy and birth, respectively, were significantly higher and the gestational age significantly lower in the I-GDM group, compared to D-GDM and controls (Table 1). HbAC1 levels were also significantly higher in I-GDM than in D-GDM women, but the majority of samples were still in the normal range. Eight of 61 (13%) women with I-GDM had diabetes before pregnancy. Thus, metabolic disturbances appear to be more pronounced in women requiring insulin treatment.

**Fig. 1:**
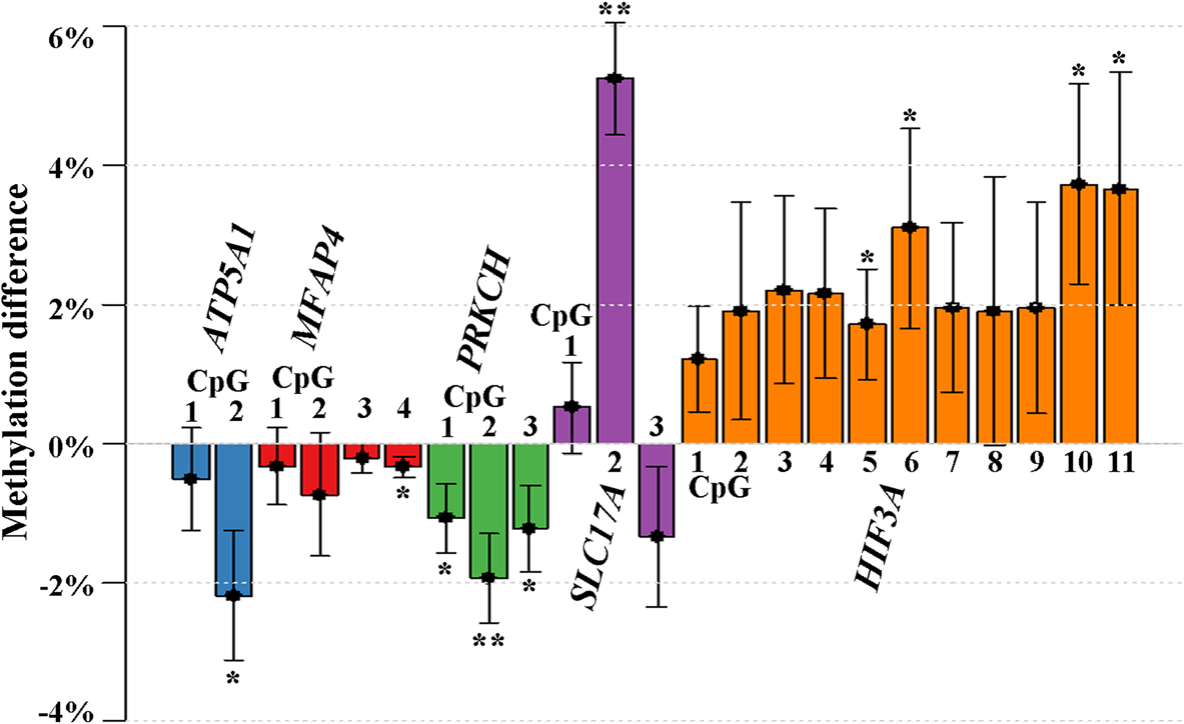
Methylation difference in fetal cord blood of GDM versus non-GDM pregnancies. Differences in methylation percentages between GDM and control FCBs are shown per CpG site for each gene studied (*ATP5A1*, *blue bars*; *MFAP4*, *red bars*; *PRKCH*, *green bars*; *SLC17A*, *mauve bars*; *HIF3A*, *orange bars*), adjusted for maternal BMI, gestational week, and fetal sex. Significant sites are indicated by *star symbols* (*p < 0.05; **p < 0.005)

The pyrosequencing assay for *ATP5A1* targeted two CpG sites in the promoter region. The array CpG (CpG2 of the pyrosequencing assay) displayed a significant methylation difference (*β* = −2%; *p* = 0.014) between GDM and control samples. When comparing I-GDM versus controls, CpG1 and the mean of both CpGs were significantly (*p* = 0.001 and 0.007) different between groups. The comparison of D-GDM versus controls did not reveal significant results. Similarly, four CpGs were analyzed in the *MFAP4* promoter-flanking region; however, neither individual CpG nor mean methylation differed between GDM and control group. The only significant difference was observed for CpG4 between I-GDM and control samples (*β* = -0.4%; *p* = 0.048). The *PRKCH* assay targeted three CpGs in an enhancer region. Each individual CpG (CpG3 being the array CpG) and their mean methylation were significantly hypomethylated (*β* = −1.1 to −1.9%; *p* < 0.005) in GDM samples, compared with controls. The same was true (*p* < 0.001) when comparing I-GDM samples versus controls. A weaker effect was seen for CpG1 (*p* = 0.034), CpG2 (*p* = 0.003), and mean methylation of all CpGs (*p* = 0.015) in D-GDM versus controls. Three CpGs were analyzed in *SLC17A4*. Consistent with the methylation screen, the array CpG (CpG3 of the pyrosequencing assay) was hypomethylated (*β* =-0.8% to -2.0%) in GDM, I-GDM, and D-GDM samples, but the results were not significant. Surprisingly, CpG2, which is 141 bp upstream of CpG3, was significantly (*p* < 0.001) hypermethylated (*β* = 4.4–5.2%) in GDM, I-GDM, and D-GDM, compared with controls (Fig. 1). Thus, the methylation difference between CpG2 and CpG3 was 4.5–6.5 percentage points (*p* < 0.001) higher in the GDM, I-GDM, and D-GDM groups than in controls.

Previously, *HIF3A* methylation in adult blood was positively correlated with BMI [49] and adipose tissue dysfunction [50]. Although it was not among the top candidate genes in our methylation screen, *HIF3A* was also analyzed by bisulfite pyrosequencing, targeting 11 CpGs in the *HIF3A* promoter (array CpGs 6, 8, and 11). Mean methylation of all 11 CpGs was significantly higher between GDM and controls (*β* = 1.3%; *p* = 0.001), I-GDM and controls (*β* = 1.5%; *p* = 0.005), and D-GDM and controls (*β* = 1.2%; *p* = 0.002). At the individual CpG level, nine of 11 CpGs were significantly hypermethylated in GDM and six of 11 in I-GDM and D-GDM samples, respectively. CpGs 3, 4, 5, 10, and 11 were significant in all three between-group comparisons, CpG 2 and 8 in none of the comparisons.

After adjusting for the potential confounding factors maternal BMI, gestational week, and fetal sex in our multivariate regression analysis (Additional file 4: Table S3), GDM was associated with significant FCB methylation changes of CpG2 (*β* = −2.2%; *p* = 0.02) in *ATP5A1*, of CpG4 (*β* = −0.4%; *p* = 0.04) in *MFAP4*, of CpG1 (*β* = −1.1%; *p* = 0.03), CpG2 (*β* = −1.9%; *p* = 0.003), and CpG3 (*β* = −1.2%; *p* = 0.05) in *PRKCH*, of CpG2 (*β* = 5.3%; *p* < 0.001) in *SLC17A4*, and of CpG5 (*β* = 1.7%; *p* = 0.03), CpG6 (*β* = 3.1%; *p* = 0.03), CpG10 (*β* = 3.7%; *p* = 0.01), and CpG11 (*β* = 3.7%; *p* = 0.03) in *HIF3A*. Mean methylation of all CpGs in the target region was significant for *PRCHK* (*β* = −1.4%; *p* = 0.008), *SLC17A* (*β* = 1.5%; *p* = 0.03), and *HIF3A* (*β* = 2.3%; *p* = 0.05).

## Discussion

The prevalence of GDM and maternal obesity is constantly increasing worldwide and gives rise to a vicious cycle in which babies exposed to GDM in utero are more likely to develop metabolic (and other) disorders later in life [12–16]. The mechanisms increasing the risk for long-term morbidity in the offspring are still poorly understood, but epigenetics is thought to be a key player in this process [18–20]. A growing number of studies in human postpartum tissues [26–36] have demonstrated GDM-related changes in the offspring’s DNA methylation patterns. In the mouse model, there is evidence that epigenetic changes in the germ cells of offspring from diabetic/obese mothers may contribute to transgenerational inheritance of a metabolic phenotype [51, 52].

The observed GDM-associated epigenetic changes in cord blood and/or placenta are small (in the order of a few percentage points) at the single-gene level but appear to be widespread. Nevertheless and similar to the hits of genome-wide association studies (GWAS), despite small effect size, the identified differentially methylated loci may uncover genes that are essential for fetal programming of a metabolic phenotype in GDM offspring. Considering the enormous variation of DNA methylation patterns among non-exposed neonates/infants, the measured methylation values in GDM offspring are still in the normal range and, thus, their diagnostic or prognostic value is currently too low for clinical implementation. Again similar to GWAS, the development of polygenic risk scores may allow better predictions of the outcome of adverse intrauterine exposures. When interpreting the functional relevance of epigenetic markers, it is important to emphasize that the epigenomes differ between cell types and tissues. Alterations in cord blood DNA methylation cells do not necessarily reflect alterations in the organs (pancreatic islets, fat, liver, skeletal muscle, and hypothalamus) that play a role in the pathogenesis of GDM. Due to ethical and legal restrictions, the target tissues for fetal programming of metabolic disease in GDM-exposed offspring are not accessible.

Moreover, there are numerous confounding factors on the maternal and offspring’s side. Differences in ethnicity (genetic background), comorbidities, diagnostic criteria for GDM, and treatment during pregnancy may explain the huge discrepancies in the number of genome-wide significant hits in conceptually very similar 450K methylation array studies [32, 35]. To the extent possible, we tried to minimize the effects of ethnicity and comorbitidies. More than 90% of our study participants from a single big obstetric clinic were Caucasians. Only a few women in each cohort/subgroup suffered from hypertension, preeclamsia, thyroid dysfunction, or other medical problems. In addition, there were only few preterm births and babies with SGA or LGA. Typical for the situation in Germany, diabetes in our GDM cohorts was very well controlled. Most GDM mothers exhibited HbA1c values in the normal range (5.5 ± 0.3% in D-GDM and 5.8 ± 0.4% in I-GDM), which may explain the relatively low number of differentially methylated CpGs in exposed offspring, compared to a recent study on South Asian pregnant women [35]. It seems plausible to assume that an early diagnosis and optimum treatment of GDM reduces epigenetic effects due to adverse intrauterine exposure. In general, we observed more significant effects in I-GDM than in D-GDM. This may be due to epigenetic effects of insulin itself or, more likely, to a more severe phenotype in women requiring insulin treatment. Ten to 17% of women in the analyzed I-GDM subgroups (but none with D-GDM) have been diagnosed with diabetes before pregnancy, consistent with an adverse environmental exposure of the embryo/fetus during early development. Maternal BMI and HbA1c levels were significantly higher in pregnant women with I-GDM, compared to D-GDM, whereas gestational age at birth was lower. In addition to maternal BMI and gestational week, fetal sex-dependent endocrine effects may play an important role in the pathogenesis of GDM [53]. However, following adjustment for the maternal BMI, gestational week, and fetal sex in a multivariate regression model, the GDM effect on the methylation patterns of the four analyzed candidate genes remained significant. This argues in favor of the robustness of our approach and the quality of our array data. In addition, there were no detectable differences in cell composition of FCB and control bloods, which could explain the observed effects.

Although the number of GDM and control samples analyzed here meets current standards for genome-wide methylation studies, the sample size is still two orders of magnitude lower than that of recent GWAS for complex phenotypes. Therefore, existing methylation array data sets are likely still polluted with false positives and false negatives. Overall, we identified 65 GDM-associated CpG methylation changes. The 55 associated genes are mainly novel and reliable candidates for fetal programming by GDM. In addition, one candidate gene, *HIF3A*, from the literature [49, 50] was validated.

*ATP5A1* encodes a subunit of mitochondrial ATP synthetase, which prevents oxidative damage by mitochondrial superoxide generation. ATP synthetase disruption by high glucose levels promotes diabetic cardiomyopathy in mouse models [47]. Genetic mutations in mitochondrial ATP synthetase cause very severe metabolic disorders, presenting as early-onset encephalo-cardiomyopathies [54]. The microfibrillar-associated protein 4 (MFAP4) is involved in cell adhesion and intercellular interactions and is highly expressed in blood vessels. Plasma MFAP4 levels have been associated with various cardiovascular complications [44] and diabetic neuropathy [55]. *PRKCH*, which is hypomethylated in GDM offspring, belongs to the protein kinase C family that is involved in diverse cellular signaling pathways. It can promote cellular senescence through transcriptional upregulation of cell cycle inhibitors p21 and p27 [56]. *PRKCH* variants have been associated with early-onset obesity [48] and increased stroke risk [45, 46]. The epigenetic regulation of the intestinal sodium/phosphate cotransporter *SLC17A4* by GDM appears to be complex. The methylation difference between two neighboring CpGs was increased, one being hypermethylated and one being hypomethylated in GDM samples. Previously, we have shown that the methylation difference between neighboring CpGs not only is due to stochastic fluctuations but also may reflect epigenetic signatures of tissue, environment, etc. [57]. Transcription factor binding site searches [58] revealed that the hypomethylated C is important to create a p53 binding site. It is tempting to speculate that the regional DNA methylation profile modulates access of transcription factors to their binding sites. A common variant near the *SLC17A4* gene has been associated with measures of atherosclerotic disease [42]. Hypoxia inducible factors (HIFs) are heterodimeric transcription factors that mediate hypoxia response in various tissues [59]. HIF3A is one of the several isoforms of the *α* subunit that can form dimers with the *β* subunit (ARNT). HIF3A plays a role in glucose and amino acid metabolism and adipocyte differentiation [60]. The increased FCB methylation in GDM offspring is consistent with an increased risk for adipositas development [49, 50].

## Conclusions

Accumulating evidence suggests that GDM leads to changes in the epigenome(s) of the exposed offspring. Since DNA methylation plays a key role in the control of gene regulation [21–23], it is plausible to assume a causal relationship between GDM-related methylation changes at births and increased disease risks in later life. Longitudinal studies on well-characterized mother-infant pairs and larger sample sizes are needed to demonstrate persistence of the epigenetic alterations into adulthood and the effect of possible (nutritional, pharmacological, and behavioral) interventions during pregnancy and postnatal period (lactation and weaning). On the long term, only meta-analyses combining genome-wide data sets generated in different laboratories with different GDM cohorts will reveal a more complete picture.

## Additional files

Additional file 1: Table S1. Primers for pyrosequencing. (DOC 52 kb)

Additional file 2: Figure S1. Estimation of blood cell composition based on 450K methylation array profiles. Blue box plots show the distribution of cell types in GDM cord blood and red box plots in control samples. The median is represented by a horizontal line. The bottom of the box indicates the 25th percentile and the top the 75th percentile. Outliers are shown as circles. (DOC 146 kb)

Additional file 3: Table S2. Global DNA methylation of different CpG island-related array CpG subsets in control, D-GDM, and I-GDM samples. (DOC 35 kb)

Additional file 4: Table S3. Multivariate analyses (adjusting for maternal BMI, gestational age, and fetal sex): CpG methylation of candidate genes in GDM versus control FCB samples. (DOC 68 kb)

## Abbreviations

BMI: Body mass index
CGI: CpG island
CpG: Cytosine phosphate guanine
D-GDM: Dietetically treated GDM
GDM: Gestational diabetes mellitus
GWAS: Genome-wide association study
HIF: Hypoxia inducible factor
I-GDM: Insulin-treated GDM
LGA: Large for gestational age
SGA: Small for gestational age
