# Supplementary material for: Epigenetic signatures of gestational diabetes mellitus on cord blood methylation
Source: Clin Epigenetics. 2017 Mar 27;9:28. doi: 10.1186/s13148-017-0329-3 (PMC5368916; doi:10.1186/s13148-017-0329-3)
Supplement: Supplementary file 1 — Primers for pyrosequencing. (DOC 52 kb) [file 13148_2017_329_MOESM1_ESM.doc]

**Additional file 1: Table S1.** Primers for pyrosequencing

| **Gene** | **Primer** | **Sequence (5’-3’)** | **Amplicon length** | **Chromosomal location**^a^  **Gene compartment** | **Number of CpGs** |
| --- | --- | --- | --- | --- | --- |
| *ATP5A1* | Forward | GGTTTGAAGTGGGGTTAAGATT | 210 bp | Chr.18: 46,098,577-46,098,787 |  |
|  | Reverse^b^ | CCCAAACCCCATCTAATACTA |  | Promoter |  |
|  | Sequencing | GTGGGGTTAAGATTTGTA |  | [ENSR00000525323](http://www.ensembl.org/Homo_sapiens/Regulation/Summary?db=core;fdb=funcgen;r=18:46098577-46098787;rf=ENSR00000525323) | 2 |
| *HIF3A* | Forward | TGGTTGAAGGGTTATTTAGGG | 227 bp | Chr.19: 46,298,269-46,298,496 |  |
|  | Reverse^b^ | ACTCTATCCCACCCCTTTT |  | Promoter |  |
|  | Sequencing 1 | TTTAGGGGGTGTAGG |  | [ENSR00001818095](http://www.ensembl.org/Homo_sapiens/Regulation/Summary?db=core;fdb=funcgen;r=19:46298269-46298496;rf=ENSR00001818095) | 7+ |
|  | Sequencing 2 | GGTGAGATGATTTTATAGGAA |  |  | 1+ |
|  | Sequencing 3 | GTTAAGAGGGGTTTTTATT |  |  | 3 |
| *MFAP4* | Forward | GGGTTTAGTTGATGTTATTGGTATAAGA | 236 bp | Chr.17: 19,384,520-19,384,756 |  |
|  | Reverse^b^ | ACTCAATTCCCTACCTCAACAA |  | Promoter flanking region |  |
|  | Sequencing 1 | GTATAAAGAGGTTTTGGTTT |  | [ENSR00001805753](http://www.ensembl.org/Homo_sapiens/Regulation/Summary?db=core;fdb=funcgen;r=17:19384520-19384756;rf=ENSR00001805753) | 2+ |
|  | Sequencing 2 | TGAGGTTGGTAAAGTG |  |  | 2 |
| *PRKCH* | Forward | TGAAAGTGGGAATTTTAATAGATATTTGTA | 258 bp | Chr.14: 61,534,229-61,534,487 |  |
|  | Reverse^b^ | CCAAACTCCCAAAAAACCTAATCTT |  | Enhancer |  |
|  | Sequencing 1 | ATATTTGTATATTTATGTTTATAGT |  | ENSR00001789871 | 2+ |
|  | Sequencing 2 | AATAGATGAATAGATAAGTAAGA |  |  | 1 |
| *SLC17A4* | Forward | GGGTTGGGGATAATTTTTTTTTAAAG | 301 bp | Chr.6: 25,779,446-25,779,747 |  |
|  | Reverse^b^ | CCAAAACTAACATCCACAAAATACA |  |  |  |
|  | Sequencing 1 | GGAAGTTAGATTTTGGG |  |  | 2+ |
|  | Sequencing 2 | GATTTTATTATGATAAGAGGAATG |  |  | 1 |

^a^ according to Ensembl release 85.

^b^ 5' end biotinylated primer.
