# Supplementary material for: Epigenetic signatures of gestational diabetes mellitus on cord blood methylation
Source: Clin Epigenetics. 2017 Mar 27;9:28. doi: 10.1186/s13148-017-0329-3 (PMC5368916; doi:10.1186/s13148-017-0329-3)
Supplement: Supplementary file 2 — Estimation of blood cell composition based on 450K methylation array profiles. Blue box plots show the distribution of cell types in GDM cord blood and red box plots in control samples. The median is represented by a horizontal line. The bottom of the box indicates the 25th percentile and the top the 75th percentile. Outliers are shown as circles. (DOC 146 kb) [file 13148_2017_329_MOESM2_ESM.doc]

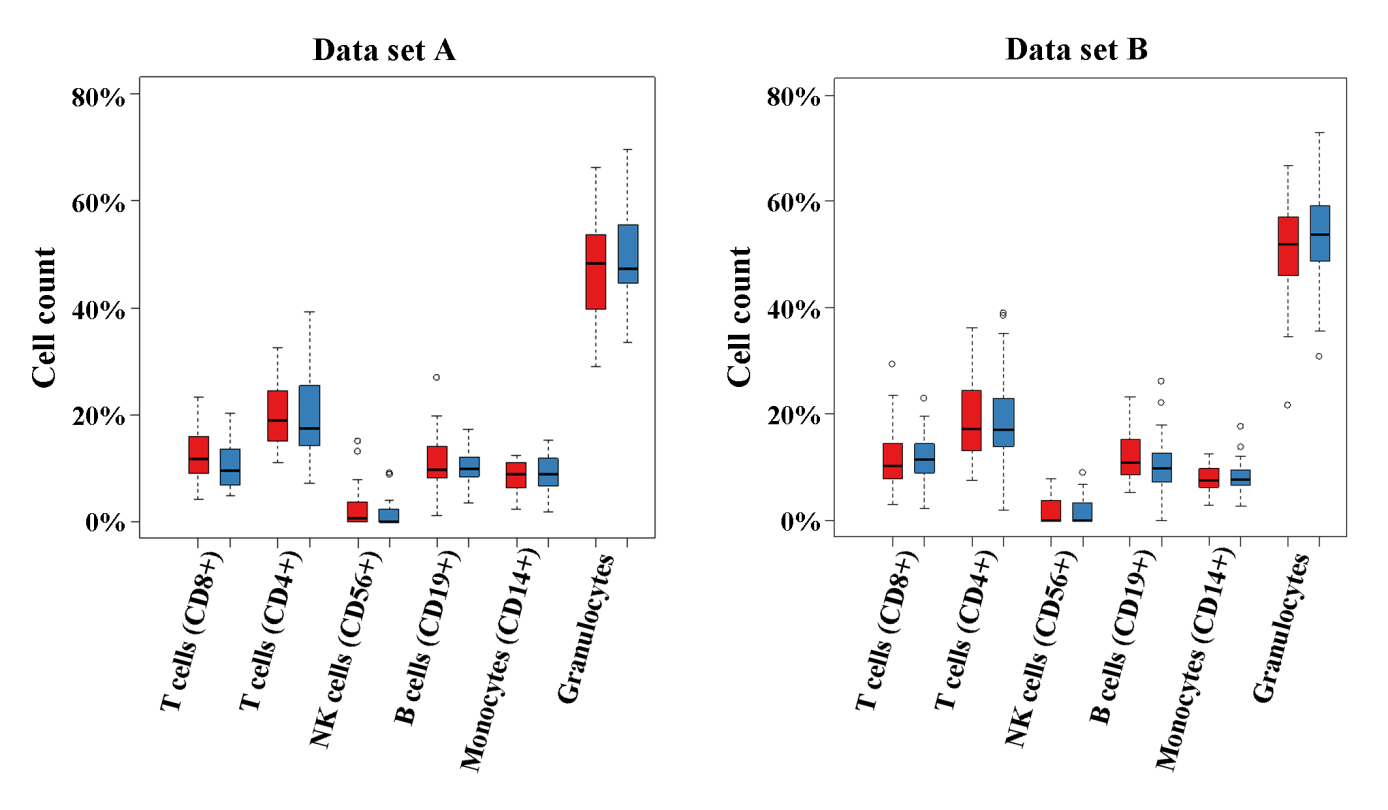


**Additional file 2: Figure S1**. Estimation of blood cell composition based on 450K methylation array profiles. Blue box plots show the distribution of cell types in GDM cord blood and red box plots in control samples. The median is represented by a horizontal line. The bottom of the box indicates the 25^th^ percentile, the top the 75^th^ percentile. Outliers are shown as circles
