# Supplementary material for: Epigenetic signatures of gestational diabetes mellitus on cord blood methylation
Source: Clin Epigenetics. 2017 Mar 27;9:28. doi: 10.1186/s13148-017-0329-3 (PMC5368916; doi:10.1186/s13148-017-0329-3)
Supplement: Supplementary file 3 — Global DNA methylation of different CpG island-related array CpG subsets in control, D-GDM, and I-GDM samples. (DOC 35 kb) [file 13148_2017_329_MOESM3_ESM.doc]

**Additional file 3: Table S2.** Global DNA methylation of different CpG island related array CpG subsets in control, D-GDM, and I-GDM samples.

|  | **Array cohort A** | | | **Array cohort B** | | | |
| --- | --- | --- | --- | --- | --- | --- | --- |
|  | **Mean methylation** | | ***p* value** | **Mean methylation** | | | ***p* value** |
|  | **Control** | **I-GDM** |  | **Control** | **D-GDM** | **I-GDM** |  |
| **CpG island** | 21.9% | 21.9% | 0.59 | 22.3% | 22.0% | 22.0% | 0.72 |
| **North shelf** | 77.1% | 77.1% | 0.94 | 76.9% | 76.2% | 76.3% | 0.16 |
| **North shore** | 47.6% | 47.6% | 0.90 | 48.1% | 47.0% | 47.0% | 0.55 |
| **South shelf** | 77.6% | 77.6% | 0.83 | 77.4% | 76.7% | 76.7% | 0.12 |
| **South shore** | 46.8% | 46.8% | 0.63 | 47.3% | 46.3% | 46.3% | 0.65 |
| **Open sea** | 73.0% | 73.0% | 0.61 | 73.0% | 72.0% | 72.0% | 0.94 |
