# Supplementary material for: Epigenetic signatures of gestational diabetes mellitus on cord blood methylation
Source: Clin Epigenetics. 2017 Mar 27;9:28. doi: 10.1186/s13148-017-0329-3 (PMC5368916; doi:10.1186/s13148-017-0329-3)
Supplement: Supplementary file 4 — Multivariate analyses (adjusting for maternal BMI, gestational age, and fetal sex): CpG methylation of candidate genes in GDM versus control FCB samples. (DOC 68 kb) [file 13148_2017_329_MOESM4_ESM.doc]

**Additional file 4: Table S3.** Multivariate analyses (adjusting for maternal BMI, gestational age, and fetal sex): CpG methylation of candidate genes in GDM versus control FCB samples

| **Gene** | **CpG site** | **ß** | **SE** | ***p* value** | **CI (low)** | **CI (high)** |
| --- | --- | --- | --- | --- | --- | --- |
| *ATP5A1* | CpG1 | -0.508 | 0.737 | 0.492 | -1.963 | 0.948 |
| *ATP5A1* | CpG2 | -2.182 | 0.934 | 0.021 | -4.025 | -0.338 |
| *ATP5A1* | CpGs1-2 | -1.345 | 0.771 | 0.083 | -2.868 | 0.177 |
| *HIF3A* | CpG1 | 1.219 | 0.768 | 0.114 | -0.297 | 2.735 |
| *HIF3A* | CpG2 | 1.905 | 1.559 | 0.223 | -1.172 | 4.983 |
| *HIF3A* | CpG3 | 2.216 | 1.355 | 0.104 | -0.459 | 4.891 |
| *HIF3A* | CpG4 | 2.165 | 1.218 | 0.077 | -0.240 | 4.570 |
| *HIF3A* | CpG5 | 1.719 | 0.799 | 0.033 | 0.142 | 3.296 |
| *HIF3A* | CpG6 | 3.101 | 1.441 | 0.033 | 0.257 | 5.945 |
| *HIF3A* | CpG7 | 1.953 | 1.220 | 0.111 | -0.455 | 4.360 |
| *HIF3A* | CpG8 | 1.903 | 1.937 | 0.327 | -1.922 | 5.727 |
| *HIF3A* | CpG9 | 1.956 | 1.522 | 0.201 | -1.049 | 4.962 |
| *HIF3A* | CpG10 | 3.737 | 1.449 | 0.011 | 0.875 | 6.598 |
| *HIF3A* | CpG11 | 3.666 | 1.677 | 0.030 | 0.355 | 6.976 |
| *HIF3A* | CpGs1-11 | 2.315 | 1.168 | 0.049 | 0.008 | 4.622 |
| *MFAP4* | CpG1 | -0.321 | 0.554 | 0.563 | -1.414 | 0.773 |
| *MFAP4* | CpG2 | -0.730 | 0.891 | 0.414 | -2.490 | 1.030 |
| *MFAP4* | CpG3 | -0.211 | 0.196 | 0.283 | -0.599 | 0.176 |
| *MFAP4* | CpG4 | -0.337 | 0.159 | 0.036 | -0.651 | -0.022 |
| *MFAP4* | CpGs1-4 | -0.411 | 0.342 | 0.232 | -1.087 | 0.265 |
| *PRKCH* | CpG1 | -1.073 | 0.489 | 0.030 | -2.039 | -0.107 |
| *PRKCH* | CpG2 | -1.932 | 0.644 | 0.003 | -3.204 | -0.661 |
| *PRKCH* | CpG3 | -1.228 | 0.614 | 0.047 | -2.439 | -0.017 |
| *PRKCH* | CpGs1-3 | -1.372 | 0.512 | 0.008 | -2.382 | -0.363 |
| *SLC17A* | CpG1 | 0.520 | 0.654 | 0.428 | -0.771 | 1.811 |
| *SLC17A* | CpG2 | 5.248 | 0.817 | 0.000 | 3.634 | 6.861 |
| *SLC17A* | CpG3 | -1.335 | 1.009 | 0.188 | -3.326 | 0.657 |
| *SLC17A* | CpGs1-3 | 1.452 | 0.677 | 0.033 | 0.116 | 2.789 |

Standard error (SE), 95% confidence intervals (CI), and the model based estimate of the methylation difference (β) are given. Significant CpGs are highlighted in red.
